# Chimeric Antigen Receptor T-Cell Revolution Remodeling Immunity to Conquer Autoimmune Disease

**DOI:** 10.34133/research.1239

**Published:** 2026-05-18

**Authors:** Wenxia Shao, Jiayu Liu, Ming Sun, Daojun Yu

**Affiliations:** ^1^ Affiliated Hangzhou First People’s Hospital, School of Medicine, Westlake University, Hangzhou 310006, China.; ^2^ School of Medical Technology and Information Engineering, Zhejiang Chinese Medical University, Hangzhou 310053, China.; ^3^ Department of Oncology, Suzhou Cancer Center Core Laboratory, The Affiliated Suzhou Hospital of Nanjing Medical University, Suzhou Municipal Hospital, Gusu School, Nanjing Medical University, Suzhou 215001, China.

## Abstract

Chimeric antigen receptor T (CAR-T) cell therapy, a groundbreaking technology in tumor immunotherapy, has demonstrated unprecedented potential in the field of autoimmune diseases in recent years. This article provides a systematic review of the developmental trajectory and core concepts of CAR-T-cell therapy in autoimmune diseases, emphasizing its conceptual evolution from traditional “killing” strategies to “precision immune remodeling”. Leveraging multitarget approaches (e.g., CD19 and B-cell maturation antigen) and chimeric autoantigen receptor technology, it achieves efficient elimination of pathogenic B cells, plasma cells, and autoreactive T cells, along with profound remodeling of the immune microenvironment, thereby inducing long-term disease remission and restoring immune tolerance. Nevertheless, unresolved challenges still exist in monotherapy strategies, such as antigen escape, nontumor toxicity of emerging targets, limited in vivo persistence, high production costs, and immune reconstitution imbalance. Future research ought to concentrate on the development of multitarget/logic-gated chimeric antigen receptor constructs, the optimization of chimeric antigen receptor architecture and nonviral delivery systems, the validation of the long-term safety of universal CAR-T cells, the customization of personalized treatment regimens, and the exploration of mechanisms for modulating the immune microenvironment. This review emphasizes that CAR-T-cell therapy shows potential for initiating a new era of personalized, mechanism-driven treatment for autoimmune diseases, offering crucial insights for clinical translation.

## Introduction

Autoimmune diseases arise from the breakdown of immune tolerance, leading to aberrant T-cell activation and the production of autoantibodies by self-reactive B cells, which cause tissue damage. Conventional therapies, including broad-spectrum immunosuppressants and CD20-targeting antibodies, often fail to eliminate pathogenic cells deeply and durably, resulting in limited efficacy, substantial side effects, and frequent relapses. In recent years, chimeric antigen receptor T (CAR-T) cell therapy, a breakthrough in tumor immunotherapy, has shown unprecedented potential in autoimmune diseases. Moving beyond a simple “killing” strategy, CAR-T cells enable “precision immune remodeling” by targeting surface antigens such as CD19 and B-cell maturation antigen (BCMA) to deeply deplete autoreactive B cells and plasma cells, reshape the immune microenvironment, and restore long-term immune tolerance. Emerging approaches—dual-target chimeric antigen receptors (CARs), chimeric autoantibody receptor T (CAAR-T) cells , and CAR-regulatory T (Treg) cells—further enhance specificity and safety. This review systematically outlines the developmental trajectory, core mechanisms, clinical efficacy, and challenges of CAR-T-cell therapies in autoimmune diseases and discusses next-generation technological frontiers to guide clinical translation.

## Development Trajectory and Core Concepts of CAR-T-cell Therapy in Autoimmune Diseases

CAR-T cells recognize and bind to antigens via antibody fragments in a major histocompatibility complex (MHC)-unrestricted manner, eliminating tumor cells. Their success in treating cancer, especially B-cell malignancies, underpins their application in treating autoimmune diseases [1]. Autoimmune pathology involves immune tolerance breakdown, leading to aberrant T-cell activation and autoantibody production by self-reactive B cells [2]. Conventional therapies often fail to deeply deplete B cells in tissues, requiring long-term treatment with limited efficacy and side effects [3,4]. For example, in systemic lupus erythematosus (SLE), anti-CD20 antibodies miss CD20-negative plasma cells [5]. CAR-T cells shift from “killing” to “precision immune remodeling” by eliminating pathogenic immune cells and restoring tolerance [2,6,7]. By targeting CD19 on B cells and plasmablasts, CAR-T cells deeply deplete autoreactive B cells in circulation and tissues, outperforming antibody therapies [5,8]. In refractory SLE, CD19 CAR-T cells rapidly deplete B cells, reduce autoantibody levels, improve symptoms, and maintain drug-free remission after B-cell reconstitution [5]. Similarly, in diseases such as idiopathic inflammatory myositis and systemic sclerosis (SSc), CD19 CAR-T cells clear disease-associated B cells and restore naive subsets, remodeling the immune microenvironment [2].

Precision immune reprogramming denotes the highly accurate and targeted modulation and reprogramming of the immune system via advanced biotechnologies, including CAR-T-cell therapy, gene editing, and nanomedicine delivery systems [9]. This methodology transcends simple functional restoration, aiming to attain the “redesign” and “functional reconfiguration” of immune responses by precisely identifying and regulating specific cell subpopulations, molecular pathways, or the immune microenvironment. With further research, the concept of “precision immune remodeling” has further expanded, manifested in the diversification of targets and the refinement of strategies. In addition to targeting CD19, CAR-T cells targeting BCMA on the surface of long-lived plasma cells, on the basis of their experience in the treatment of multiple myeloma, have been applied to diseases such as neuromyelitis optica spectrum disorder (NMOSD) and immune-mediated necrotizing myopathy (IMNM), reducing pathogenic plasma cells to decrease autoantibody levels and induce remission [10,11]. To overcome the limitations of single-target approaches, combined targeting strategies have emerged. Examples include CD19/BCMA dual-target CAR-T cells in refractory SLE, which simultaneously deplete B cells and plasma cells, affecting a dual reset of humoral immunity and the B-cell system. All treated patients achieved a low disease activity state [12,13]. Furthermore, CAAR-T cells, such as desmoglein 3 (DSG3)-CAAR-T cells (targeting pemphigus vulgaris [PV] anti-DSG3 autoantibodies) and muscle-specific tyrosine kinase (MuSK)-CAAR-T cells (targeting myasthenia gravis [MG] anti-MuSK autoantibodies), identify pathological B-cell receptors (BCRs) to eliminate specific populations of autoreactive B cells, thereby minimizing the impact on normal B cells and enhancing targeting precision [14,15]. In addition to eliminating pathogenic effector B cells, the immunomodulatory function of CAR-T cells constitutes a pivotal component of “precision immune remodeling”. CAR-engineered Treg cells can specifically recognize self-antigens (e.g., myelin oligodendrocyte glycoprotein in multiple sclerosis) and localize to pathological sites, where they exert stable immunosuppressive effects while avoiding the risks of functional instability or transdifferentiation inherent to conventional Treg cells. In animal models, they effectively mitigate inflammation and prevent relapse [16,17]. This paradigm shift from cytotoxic “killing” to immunomodulatory “regulation” presents another therapeutic paradigm for autoimmune diseases. Safety and durability are paramount considerations in “precision immune remodeling”.

Long-term follow-up demonstrated sustained remission following a single infusion. Notably, disease relapse did not occur in some patients even after B-cell reconstitution, potentially attributable to immune repertoire reset (characterized by reduced memory B cells and restored naive B cells) and reestablished immune tolerance [8,9,18]. Current applications of CAR-T cells in autoimmune diseases continue to evolve, encompassing universal allogeneic CAR-T cells (mitigating immune rejection risk via gene editing) [1,19], multitarget CAR designs, and derivative technologies such as CAR-Treg cells and CAAR-T cells, all of which aim to increase the precision, safety, and accessibility of treatment [17]. These advancements confirm the shift in CAR-T cells from a tumor “killing” strategy to the concept of “precision immune remodeling” for autoimmune diseases, providing innovative therapeutic approaches for refractory autoimmune diseases [2,3,7]. Table 1 lists the mechanisms of action and clinical efficacy of representative CAR-T-cell therapies in autoimmune diseases.

**Table 1.** Mechanisms and clinical efficacy of CAR-T-cell therapy in autoimmune diseases

Disease type	Type of CAR	Therapeutic mechanism	Therapeutic efficacy
Refractory systemic lupus erythematosus (rSLE)	CD19 CAR-T, CD19/BCMA dual-target CAR-T	Eliminates circulating or tissue-resident CD19^+^ B cells and plasmablasts; reduces the secretion of autoantibodies; restores complement levels and achieves immune “reset”.	Anti-CD19 CAR-T cell lowers autoantibody titers, normalizes complement, reduces SLEDAI scores, and achieves durable remission with naive-B-cell repopulation. This remission can be long-lasting as the longest disease-free observation period is now 22 months [83].
	Autologous anti-CD19 and anti-BCMA CAR-T cells (infused in multiple doses, non-bispecific CAR)	Eliminate peripheral CD19^+^ B cells and bone marrow CD19^-^BCMA^+^ long-lived plasma cells, reestablish an immune landscape dominated by naive B cells, and down-regulate interferon and BAFF signaling.	A median follow-up of 712 d showed no dose-limiting toxicity. Disease remission was achieved in 80% of patients at 12 weeks, with 12 cases attaining a SLEDAI-2K score of zero. Autoantibodies decreased substantially, and pathogenic B-cell clones were completely eradicated [12].
Autoimmune hemolytic anemia (AIHA) [84]	Autologous CAR-T cells targeting CD19 (murine single-chain variable fragment, 4-1BB costimulatory domain)	Deeply eliminate CD19-positive B cells, including tissue-resident autoreactive B cell clones, thus eliminating the source of antierythrocyte antibody production and restoring immune balance.	All 11 patients achieved complete remission, with a median time to remission of 45 d and a median duration of remission without medication of 11.5 months. Safety was manageable, primarily involving grade 1 to 2 CRS. Two patients who relapsed achieved remission again after receiving BCMA-targeted therapy.
Refractory myasthenia gravis (MG) [85]	Autologous BCMA/CD19 dual-targeted CAR-T cell(s)	Eliminate B cells and plasma cells, induce B-cell immune reset, and reveal that FCRL5^+^ age-related B cells represent a novel mechanism for recurrence.	Five patients achieved drug-free remission at 6 months and maintained it for 12 months without medication or symptoms; 3 patients developed negative AChR antibodies. Safety was favorable, with only 2 cases of grade 1 CRS. This therapy validated the immune reset concept and, for the first time, revealed FCRL5^+^ ABCs as a novel mechanism of MG recurrence and a potential therapeutic target.
Neuromyelitis optica spectrum disorder (NMOSD) [10]	Anti-BCMA CAR-T cell	Targeted plasma cell depletion and disruption of the pathogenic humoral immune response via CAR-mediated cytotoxicity lead to clearance of AQP4-IgG-producing cells and attenuation of downstream inflammatory cascades.	All 12 patients exhibited a decreasing trend in AQP4-IgG antibody levels after treatment, and 11 patients had undetectable serum levels during follow-up. Over a median follow-up period of 5.5 months, 11 out of 12 (92%) patients remained free of disease recurrence.
Systemic sclerosis (SSc) [86]	CD19 CAR-T cell	Pathogenic B cells secrete pro-fibrotic cytokines, such as TGF-β, which drive fibroblast activation and collagen deposition. CAR-T-cell therapy eliminates these cells, halting fibrosis and mitigating tissue damage caused by Breg imbalance.	Progression of pulmonary fibrosis in SSc patients was effectively halted. Treatment efficacy was proven to be durable, with a median follow-up duration of 15 months. Patients maintained long-term drug-free remission, and autoantibody levels remained low or negative throughout the extended follow-up [9].
Idiopathic inflammatory myositis (IIM)	CD19 CAR-T cell	Eliminate pathogenic B cells, reduce the levels of proinflammatory autoantibodies, and suppress T-cell-mediated muscle destruction.	In a CAR-T therapy study for patients with severe IIM, all 3 patients achieved ACR-EULAR major clinical remission and successfully discontinued treatment. No significant relapse occurred during the follow-up periods ranging from 15 to 29 months [9].
Rheumatoid arthritis (RA)	CD19 CAR-T cell, TNF-α-CAAR-T cells, citrullinated peptide-targeted CAAR-T cells	CAR-T-cell therapy eliminates B cells that produce rheumatoid factor and T cells specific to citrullinated peptides, thereby halting the inflammatory cascade, macrophage activation, and subsequent pannus formation.	A 48-year-old male patient with concurrent RA and MG experienced significant improvement in RA symptoms after 3 months of treatment, accompanied by a decrease in anti-cyclic citrullinated peptide (anti-CCP) antibody levels [87].

AQP4, aquaporin-4; IgG, immunoglobulin G; Breg, regulatory B cell; TGF-β, transforming growth factor-β; CRS, cytokine release syndrome; SLEDAI-2K, SLE Disease Activity Index; CAR-T, chimeric antigen receptor T; TNF-α, tumor necrosis factor-α; BCMA, B-cell maturation antigen; BAFF , B-cell activating factor; FCRL5^+^, Fc receptor-like 5-positive; AChR, acetylcholine receptor; ABC, age-associated B cell; ACR-EULAR, American College of Rheumatology/European League Against Rheumatism

## In-depth Analysis of Efficacy Mechanisms: The Biological Basis of Immune Remodeling

CAR-T-cell therapy eradicates pathogenic B cells by targeting CD19/BCMA, offering a potent treatment for autoimmunity. These B cells promote disease progression through autoantibody production, interaction with other immune cells, and antigen presentation. By rapidly and deeply eradicating B cells, CAR-T-cell therapy can dismantle the follicular architecture within germinal centers (GCs) and reconstitute the entire B-cell immune repertoire. Treatment achieves profound, long-term B-cell depletion, with immune reconstitution dominated by naive, nonautoreactive B cells, thereby restoring immune tolerance. Optimized CAR architectures—incorporating dual-targeting strategies and refined spacers—substantially increase therapeutic depth and durability, establishing a mechanistic foundation for sustained remission in refractory autoimmune diseases.

### Pathogenic role of B cells in autoimmune diseases and their immunopathological mechanisms

**Fig. 1.**
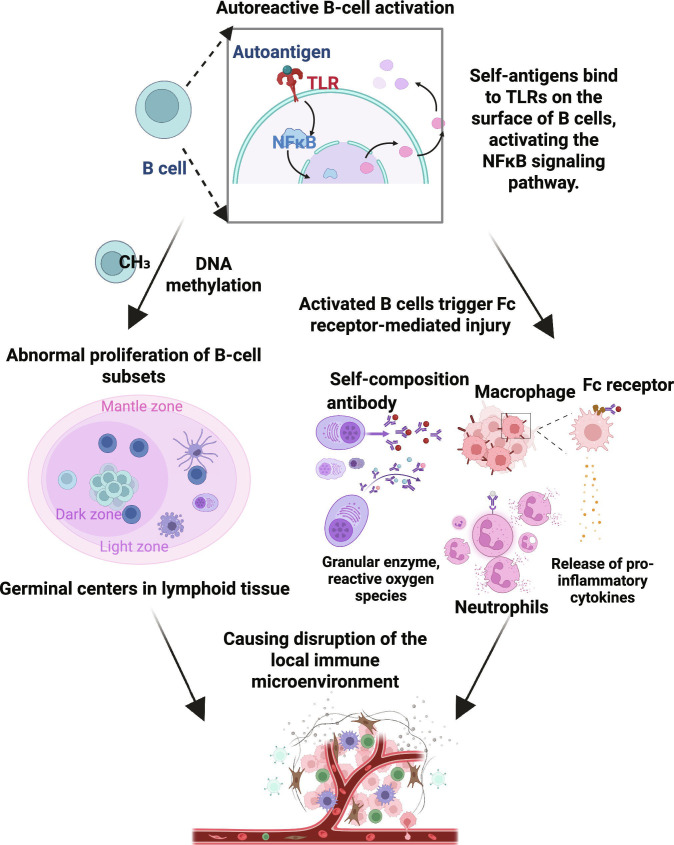
Mechanism of autoreactive B-cell activation and linked immune-mediated pathological processes. This diagram outlines autoreactive B-cell activation and its pathological effects. Autoantigens bind to Toll-like receptors (TLRs) on B cells, activating the nuclear factor κB (NFκB) signaling pathway. DNA methylation drives the dysregulated expansion of B cells in germinal centers. Activated B cells produce autoantibodies that engage Fc receptors on macrophages/neutrophils, triggering the release of granzymes, reactive oxygen species (ROS), and proinflammatory cytokines, which causes tissue damage. These processes alter the immune microenvironment, sustaining the dysregulation. Created in BioRender.com.

In autoimmune diseases, B cells contribute to pathology through a 4-step mechanism: activation of autoreactive B cells that produce antibodies, immune complex-mediated inflammation, antigen presentation-activating T cells, and regulation of the local immune microenvironment [20]. SLE is characterized by autoantibody- and immune complex-mediated organ damage, with autoreactive B cells being pivotal; in lupus nephritis (LN), the clonal expansion and somatic hypermutation of B cells in renal tubulointerstitial T:B aggregates/GCs suggest local antigen-driven disease progression [21]. Autoantibodies are critical in multiple autoimmune diseases: In MG/Lambert–Eaton syndrome, antibodies attack neuromuscular junctions, causing conduction disorders [22]; in NMOSD, plasma cell-derived anti-aquaporin-4 (AQP4) immunoglobulin G (IgG) triggers pathology; and in type 1 diabetes, B-cell antibodies increase antigen presentation, driving disease progression. Autoantibodies exacerbate inflammatory damage via complement activation and Fc receptor binding. B-cell antigen presentation is crucial: In type 1 diabetes models, B cells recognize self-antigens via BCRs and present them to CD4^+^ T cells, with BCR specificity determining autoimmune development; B cells lacking MHC II or with restricted BCRs can limit T-cell responses to islet antigens [23]. Additionally, epigenetic abnormalities (e.g., DNA methylation) lead to B-cell hyperactivation, and abnormal expansion of subsets such as marginal-zone B cells/GC B cells contributes to pathology; aberrant activation-induced cytidine deaminase (AID) expression in GC B cells affects BCR diversity and autoreactive B-cell formation [24]. CD20 antibodies are unable to eliminate CD20-negative plasma cells and tissue-resident B cells, which restricts their effectiveness. CD19, which is ubiquitously expressed on B cells and plasmablasts, represents an ideal target for CAR-T cells. Gene-edited allogeneic CD19 CAR-T cells can eliminate blood and tissue B cells (including skin-infiltrating B cells), reverse muscle inflammation and fibrosis, and offer evidence for tissue B-cell clearance [1]. B-cell function is associated with regulatory B-cell imbalance: B cells display a pro-inflammatory/anti-inflammatory duality, with regulatory B cells modulating immunity through interleukin-10 (IL-10), transforming growth factor-β (TGF-β), and cell contact; their dysfunction exacerbates autoimmunity [25]. CAR-T-cell-mediated profound depletion of abnormal B cells blocks autoantibody production and remodels the immune microenvironment; posttreatment reconstituted B cells are predominantly nonautoreactive naive B cells, leading to immune “reset” [2]. Although “on-target off-tumor” toxicity may occur [26], clinical data indicate favorable safety and rapid, durable remission, providing empirical support for the crucial role of B-cell clearance (Fig. 1).

### CAR-T-cell-induced B-cell and immune microenvironment-mediated mechanisms

**Fig. 2.**
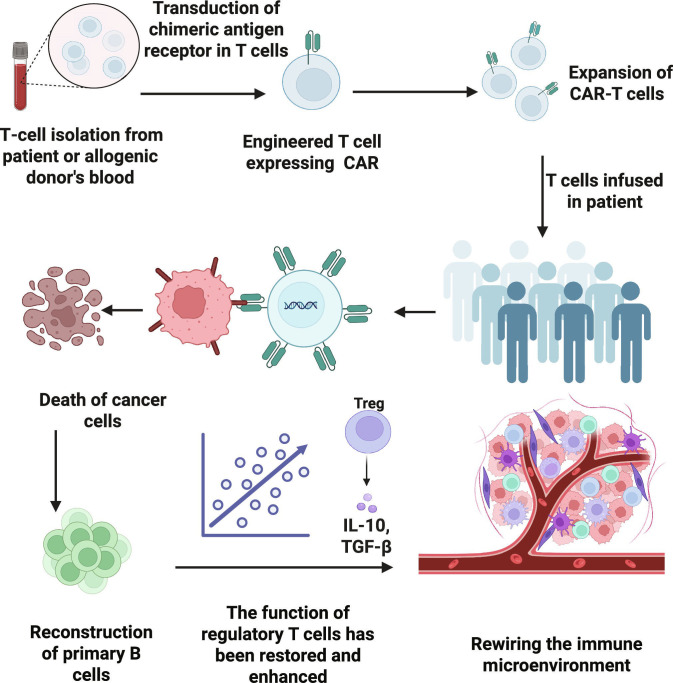
Workflow and immunomodulatory effects of chimeric antigen receptor T (CAR-T) cell therapy. This figure illustrates the CAR-T-cell therapy process and its immunomodulatory effects. T cells are isolated from patient or donor blood, engineered with a chimeric antigen receptor (CAR), expanded ex vivo, and infused to target and kill cancer cells. This therapy enhances regulatory T-cell function, increasing anti-inflammatory cytokine secretion, which promotes B-cell reconstitution and immune microenvironment rewiring for long-term immune homeostasis. Created in BioRender.com.

The CAR-T-cell therapy process involves collecting blood from the patient, isolating and activating T cells, introducing the CAR gene via viral or gene editing technology, proliferating the cells in vitro, and then reinfusing them into the patient after rigorous quality control [27].CAR-T cells eliminate pathogenic B cells by targeting B-cell surface antigens (such as CD19 and BCMA), reshaping the immune microenvironment to restore immune tolerance, which is the core mechanism mediating immune system reset. The specificity and depth of B-cell clearance determine the efficacy of immune reset. Einarsdottir et al. [28] reported that CD19 CAR-T cells induce long-term apoptosis of CD19^+^ B cells (lasting years), affecting both naive and memory B cells, whereas BCMA CAR-T cells eliminate only BCMA^+^ plasma cells and have no substantial effect on early B cells. Qin et al. [11] reported a case in which refractory IMNM patients were treated with BCMA CAR-T cells, in which CD3-CD19^+^ B cells nearly disappeared within 2 months, and the recovery phase was dominated by naive B cells with sustained anti-signal recognition particle antibody negativity. Stock et al. [29] reported that patients with CD28z-costimulated CAR-T cells experienced faster B-cell recovery, whereas 41BBz costimulation led to prolonged B-cell depletion. Immune microenvironment remodeling involves antigen status and cytokine network regulation. Lin et al. [30] developed universal CAR-T cells by using CRISPR-Cas9 to knock out TRAC and B2M, reducing graft-versus-host disease (GVHD) and immune rejection, promoting CAR-T-cell proliferation and long-term survival, and indirectly regulating the immune microenvironment. Immune tolerance restoration is closely linked to normalization of the microenvironment after B-cell clearance. Cappell et al. [31] reported that some long-term remission patients regained normal nonmalignant B cells, suggesting that the immune system was partially reset. Tur et al. [8] further confirmed that CAR-T-cell therapy leads to the disappearance of follicular dendritic cell networks and follicular helper T (TFH) cells, potentially interrupting B-cell-dependent survival signals (e.g., lymphotoxin-β maintains follicular dendritic cell survival, inducible T-cell costimulator ligand/inducible T-cell costimulator, and CD40/CD154 pathways regulating TFH cells), disrupting GC structures, and hindering autoreactive B-cell activation and differentiation. Hernani et al. [32] reported that CAR-T-cell-induced cytokine release syndrome (CRS) and immune effector cell-associated neurotoxicity syndrome (ICANS) reshape the immune microenvironment through excessive immune activation (release of IL-6, IL-1, etc.) and blood–brain barrier disruption and that blocking cytokine pathways (e.g., using tocilizumab) can modulate toxic responses, indirectly affecting B-cell clearance efficiency and immune tolerance restoration (Fig. 2).

Furthermore, the stability of Treg cells might contribute to CAR-T-cell-mediated immune remodeling, and their function is dependent on FoxP3 and specific epigenetic modifications [33]. CAR-Treg cells are an advanced immunomodulatory platform constructed by grafting antigen-specific recognition capabilities onto the surface of Treg cells through genetic manipulation [34]. The basic design principle combines the high specificity of the CAR recognition domain with the immunosuppressive function of Treg cells, with the aim of achieving a therapeutic model characterized by “precise targeting and minimal systemic immunosuppression” [35]. This approach effectively overcomes the limitations of traditional polyclonal Treg therapy, which frequently encounters issues of inaccurate homing and off-target effects due to antigen nonspecificity [36]. Via the CAR domain, CAR-Treg cells can specifically recognize diseased tissues or autoantigens such as CD19, thus precisely homing to inflammatory lesions or target organs and avoiding the adverse effects related to systemic immunosuppression. Mechanistically, CAR-Treg cells exert their regulatory function through direct cell–cell contact, which suppresses the activity of effector T cells and simultaneously secretes anti-inflammatory cytokines such as IL-10, TGF-β, and IL-35 [37]. These cytokines induce the polarization of macrophages toward the M2 phenotype, thereby remodeling the local microenvironment into a state of immune tolerance and halting the progression of inflammation. In the context of autoimmune diseases, CAR-Treg cells specifically recognize B-cell surface antigens or BCR-specific epitopes, directly inhibiting the activation and antibody production of autoreactive B cells or blocking their antigen presentation to effector T cells [38]. This intervention fundamentally disrupts the pathogenic B–T-cell interaction loop. CAR-Treg cells have exhibited distinct clinical potential in a variety of autoimmune conditions. In multiple sclerosis [39], CAR-Treg cells can cross the blood–brain barrier and home to demyelinated regions, suppress the infiltration of pathogenic T helper 1/T helper 17 cells, and promote remyelination through the secretion of neuroprotective factors such as brain-derived neurotrophic factor. In autoimmune hepatitis, CAR-Treg cells specifically recognize hepatic autoantigens, directly suppress intrahepatic autoreactive immune cells, and restore immunological homeostasis within the liver microenvironment [40]. In conclusion, by integrating “targeted homing” with “global suppression”, CAR-Treg cells not only establish a localized immune-tolerance barrier at inflammatory sites but also remodel the microenvironment and disrupt pathogenic B–T-cell interactions. This “precision immune remodeling” strategy presents a promising approach for the definitive treatment of autoimmune diseases.

## Discussion on the Breadth of the Target Lineage and Disease Applicability

Traditional CD20-targeting biologic therapies are limited by poor tissue penetration and the inability to eradicate plasma cells. CD19 CAR-T cells achieve greater depletion but fail to eliminate CD19-negative plasma cells. Emerging targets such as BCMA show promise in clearing plasma cells in NMOSD and myositis, although off-tumor toxicity and limited durability remain challenges. Autologous antigen-specific CARs (CAARs) enable precise targeting to reduce side effects, but their clinical efficacy needs to be evaluated. Target diversification—including multiple B-cell markers, CAR-Treg cells, and T-cell-specific antigens—expands CAR-T-cell applicability to neuroimmune and inflammatory disorders beyond B-cell diseases. Over 380 clinical trials are investigating multitarget strategies to overcome disease heterogeneity and prevent antigen escape.

### Applications and limitations of traditional targets CD19 and CD20

**Fig. 3.**
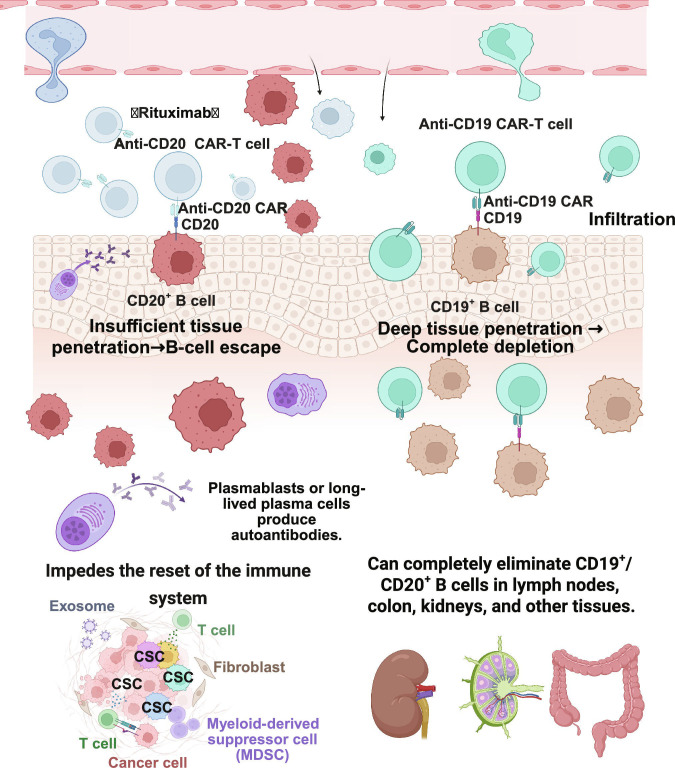
Applications and limitations of traditional targets CD19 and CD20. This figure contrasts the effects of CD20-targeted and CD19 chimeric antigen receptor T (CAR-T) cell therapies on B-cell clearance and immune regulation. The left panel shows that CD20-targeted therapies, such as rituximab, cannot fully eradicate CD20^+^ B cells because of poor tissue penetration, which allows these cells to evade elimination. Additionally, they fail to target plasmablasts/plasma cells, which persist and produce autoantibodies, disrupt the immune microenvironment, and hinder immune reset. The right panel shows that anti-CD19 CAR-T cells have a profound ability to penetrate tissues, enabling the complete elimination of CD19^+^/CD20^+^ B cells in various tissues, including the lymph nodes, colon, and kidneys, resulting in comprehensive B-cell depletion. Created in BioRender.com.

Traditional CD20-targeted therapies (such as rituximab) have limited efficacy in treating autoimmune diseases, primarily because they cannot adequately clear B cells within tissues, leading to substantial B-cell escape and hindering immune system reset [4]. In addition, CD20 is not expressed on plasmablasts or long-lived plasma cells, which continuously produce autoantibodies, making it a core issue that is difficult to eradicate through immunotherapy [5]. Additionally, after rituximab treatment, tissue-resident memory B cells in lymphoid organs and inflammatory tissues can persist, with intact lymph node follicular structures, preventing effective disruption of the immune activation cascade [4,8]. In diseases such as SLE, this results in suboptimal symptom control and B-cell depletion, highlighting its limitations [41]. In comparison, CD19 CAR-T-cell therapy targets CD19, enabling deep tissue penetration to eliminate B-cell populations, including plasmablasts, achieving profound B-cell depletion and inducing immune system “reboot”. Sustained efficacy is another advantage, with SLE patients maintaining clinical and immunological remission for up to 29 months of follow-up, accompanied by immune reconstitution characterized by reduced memory B cells and increased proportions of naive B cells, suggesting that the B-cell lineage is reset [2,18]. Tur et al. [8] reported that CD19 CAR-T cells are capable of not only eliminating B cells in the peripheral blood but also infiltrating secondary lymphoid tissues (e.g., lymph nodes), thereby thoroughly eradicating CD19^+^ and CD20^+^ B cells and disrupting TFH cell structures. This effect cannot be attained through traditional anti-CD20 monoclonal antibody therapy. B-cell clearance has also been detected in nonlymphoid tissues such as the colon, kidneys, and gallbladder, which validates their extensive tissue infiltration capacity [8]. In terms of safety, most patients experienced only mild CRS, with no severe neurotoxicity, manageable hematologic toxicity, or controlled infections. Allogeneic CD19 CAR-T-cell products (e.g., TyU19) also demonstrated effective B-cell clearance and clinical remission [1,19]. However, CD19 CAR-T cells still have several limitations: They cannot eliminate long-lived CD19-negative plasma cells, which may continuously produce certain autoantibodies [42]. Although single-target CD19 CAR-T cells have shown preliminary efficacy in SLE, combination therapy with targets such as BCMA is needed to achieve deeper B-cell depletion (Fig. 3).

### Emerging target expansion: BCMA, CD138, and autologous antigen-specific CAR

BCMA, as an emerging target, has demonstrated substantial efficacy in treating hematological malignancies, and its application in the treatment of autoimmune diseases is gradually expanding and showing unique potential. In a phase I clinical trial of patients with relapsed/refractory AQP4-IgG-positive NMOSD, the BCMA-targeted CAR-T-cell therapy CT103A exhibited manageable safety, with 92% of 12 patients achieving relapse-free status; a substantial reduction in serum pathogenic AQP4–IgG levels; and improvements in multiple functional indicators, such as disability assessment, visual ability, and quality of life [10]. With respect to antisignal recognition particle myopathy, BCMA CAR-T-cell therapy results in sustained clearance of pathogenic autoantibodies for more than 18 months, enabling B-cell lineage reconstruction and restoration to near-normal functional states while modulating the T-cell-mediated immune microenvironment and reducing proinflammatory features [11]. In SLE and LN, BCMA-CD19 composite CAR (cCAR) T cells, which simultaneously target B cells and long-lived plasma cells, achieved complete seroconversion of autoantibodies in all 12 patients within 3 months, with substantial reductions in the SLE Disease Activity Index (SLEDAI-2K). Some patients maintained remission for up to 46 months during follow-up, validating the advantage of combining BCMA and CD19 in broadening target cell clearance [13]. However, the widespread expression of BCMA poses challenges: Its presence in other normal cells and nonmalignant tissues, such as neurons and astrocytes, may lead to on-target, off-tumor toxicity, such as movement disorders and Parkinson’s disease-like symptoms. Additionally, in autoimmune disease contexts, compared with those in tumor environments, BCMA CAR-T cells exhibit distinct cellular kinetic profiles [43]. To increase specificity, researchers have developed various optimization strategies, including tuning the affinity of low-affinity single-chain variable fragments (scFvs) to recognize high-antigen-density tumor cells, logic-gated CAR designs, and synthetic Notch receptors to enable multigen-dependent activation [44].

In addition to BCMA, self-antigen-specific CARs provide a new direction for the precise treatment of autoimmune diseases. With respect to mucosal pemphigus (PV), DSG3-CAAR-T cells specifically recognizes anti-DSG3 B cells, inhibits pathogenic antibody responses in PV mouse models, reduces epithelial tissue binding, and alleviates blister symptoms, and in vitro experiments have shown that DSG3-CAAR-T cells can reduce 83.1% to 92.3% of anti-DSG3 IgG B cells without substantially affecting total IgG B cells, demonstrating high targeting specificity [14]. In MuSK MG, MuSK-CAAR-T cells specifically lyses B cells targeting different structural domains of MuSK, substantially reducing anti-MuSK IgG levels in experimental autoimmune myasthenia gravis model mice without affecting total B cells or IgG levels, and systemic off-target safety assessments have revealed no substantial toxicity. Its platform technology holds potential for extension to other targets, such as BCMA and CD138 [15]. Additionally, human leukocyte antigen (HLA)-DR1-based CAR-T cells, through HLA-DR1 molecules carrying type II collagen (CII) peptides, recognize autoimmune CD4^+^ T cells, decreasing disease incidence and severity in rheumatoid arthritis models. Their dual-chain signaling domain design offers a novel approach for targeting T-cell-mediated autoimmune diseases [45]. CD138, another potential target, has been mentioned in some studies as a direction for expansion.

### Driving role of target diversification in expanding the disease spectrum

Targeted diversification now propels CAR-T-cell therapy beyond B-cell-mediated autoimmunity into neuroimmune, inflammatory, and even autoimmune-associated oncological disorders [17]. By exploiting various surface antigens (CD19/20/22), autoantigen-engineered CAARs, or antigen-peptide-guided systems, engineered T cells can selectively delete pathogenic B-cell subsets, autoantibody-producing clones, or pathogenic Treg niches while sparing global immunity—achieving durable remission in SLE, antisynthetase syndrome, SSc [7], rheumatoid arthritis, and pemphigus models. Carcinoembryonic antigen-specific CAR-Treg cells constructed against carcinoembryonic antigen can effectively accumulate at inflammatory sites and inhibit the progression of inflammatory bowel disease and its associated colorectal cancer through the secretion of immune regulatory factors such as IL-10 [46]. Similarly, CAR-Treg cells designed for the type 1 diabetes autoantigen insulin, although not completely blocking the onset of diabetes in nonobese diabetic (NOD) mice, have the potential to expand CAR-Treg cells through autoantigen targets in specific AID applications [47]. These studies indicate that the target selection of CAR-Treg cells can be customized on the basis of disease-specific antigens, thereby broadening the therapeutic spectrum. For T-cell-mediated AID, strategies targeting self-antigen peptide–MHC complexes show promise. In 2016, Fishman et al. [48] utilized mRNA transfection technology to program autologous CD8 T cells into chimeric receptor T cells carrying self-antigen peptides such as InsB15–23 and IGRP206–214. In NOD mice, this approach substantially delayed the onset of diabetes, demonstrating that diversifying targets against pathogenic T cells specific to different self-antigens can advance the application of CAR-T cells in T-cell-mediated diseases. Additionally, CAR designs based on pMHC recognition domains can specifically eliminate pathogenic T cells, offering new strategies for treating neuroimmune disorders such as multiple sclerosis. Multitarget technology and combination strategies have further facilitated the expansion of the disease spectrum. Designs such as dual CARs, tandem CARs, trivalent CARs, and hybrid CAR-T cells can address the heterogeneity of AID and antigen escape mechanisms. Notably, the concept of multitarget combinations is not limited to treatment itself. In 2013, Rawstron et al. [49] employed a multimarker combination strategy (CD19/CD5/CD20) for minimal residual disease detection in chronic lymphocytic leukemia, providing a technical pathway reference for the selection and optimization of CAR-T-cell targets in AID, thereby advancing the in-depth understanding of disease heterogeneity. Currently, more than 380 clinical trials targeting dozens of AIDs, including SLE, SSc, and multiple sclerosis, encompass a broad exploration of target combinations and therapeutic modalities. Both autologous CAR-T cells and allogeneic CAR-T cells and multispecific antibodies are optimized for different target configurations and disease states, collectively demonstrating that target diversification is the core driver of the expansion of CAR-T-cell therapy into broader autoimmune disease areas.

## In-depth analysis of long-term efficacy, safety, and management strategies

Long-term CAR-T-cell efficacy in autoimmune diseases requires robust in vivo expansion, persistence, and thorough B-cell clearance in tissues, enabling immune reconstitution with nonautoreactive clones and autoantibody loss. Optimal dosing is crucial for durable remission. Safety management must address CRS, neurotoxicity, and chronic immune dysregulation, such as hypogammaglobulinemia, and infection risk, which can be mitigated by immunoglobulin replacement and surveillance. Prognostic assessment depends on detecting residual disease and tracking immunoglobulin levels. This integrated approach of continuous monitoring and proactive management underpins sustainable remission and a favorable therapeutic index.

### Persistence of CAR-T cells and immune reconstitution

The in vivo expansion and sustained survival of CAR-T cells constitute the core foundation for long-lasting efficacy. Immune reconstitution, defined as a dynamic recuperative process, entails the restoration of cellular competency and functional diversity within the immune system subsequent to immunosuppressive or ablative therapies [50]. Its overarching objective is to establish effective immunosurveillance and defense mechanisms while concurrently preserving immune homeostasis and tolerance. Precision immune remodeling represents a therapeutic paradigm in which pathological immune subpopulations are identified with high specificity. This facilitates the targeted eradication of disease-causing clones at the microarchitectural level and the simultaneous reconstruction of the immune microenvironment. In contrast to traditional immune reconstitution, which primarily concentrates on restoring the quantity and general functionality of immune cells, precision immune remodeling strives to eliminate fundamental pathogenic structures. Therefore, its ultimate therapeutic objective is to establish a functionally competent and self-tolerant immune system [51]. Müller et al. [9] further confirmed that the peak time for CAR-T cells was 8.6 ± 0.8 d, with a median peak level of 146 cells per microliter, demonstrating efficient expansion characteristics. In patients with relapsed/refractory B-cell acute lymphoblastic leukemia, the median duration of tisagenlecleucel (CTL019) reached 168 d, with the longest duration being 20 months [52]. Allogeneic CD19-targeted CAR-T cells (TyU19) also survive for more than 3 months in patients with refractory autoimmune diseases [1], providing critical support for the durability of therapeutic efficacy. The expansion of CAR-T cells typically precedes the clearance of circulating B cells, and the dynamic balance between the two is closely related to therapeutic efficacy. Mougiakakos et al. [41] reported that CAR-T-cell expansion occurs before the complete disappearance of B cells, and this temporal relationship is critical for sustained efficacy. Feng et al. [42] reported that in CD19/BCMA dual-target CAR-T-cell therapy for refractory SLE, peripheral blood B cells recovered approximately 3 months after treatment, whereas Müller et al. [9] reported that CD19^+^ B cells were eliminated within an average of 5.9 ± 2.2 d postinfusion, with B cells reappearing in 14/15 patients after an average of 112 ± 47 d (median, 100 d). Notably, the pretreatment regimen substantially affected this balance: Patients who received anti-CD20 monoclonal antibody therapy had lower baseline B-cell counts and longer cell-free periods (median, 138 d versus 86 d) but shorter CAR-T-cell survival times (median, 40 d versus 58 d) [9]. Additionally, B-cell recovery coincides with a decrease in the number of CAR-T cells, and the newly generated B cells are predominantly nonautoreactive clones, with immunoglobulin diversity similar to that of healthy individuals [2]. Wang et al. [13] further confirmed through BCR deep sequencing that immune system reconstruction after BCMA-CD19 cCAR-T-cell therapy is dominated by nonclass-switched IgM heavy chains (>95%), with the absence of IgG and IgA clonotypes, resulting in a functional reset of the B-cell population from memory to naive types. Immune reconstruction mediated by CAR-T cells can be intuitively reflected through dynamic changes in autoantibodies, complement, and immunoglobulins. Mougiakakos et al. [41] reported that a patient’s double-stranded DNA antibody concentration decreased from more than 5,000 to 4 U/ml (negative) within 5 weeks, accompanied by normalization of C3 and C4 complement levels, indicating a reversal of immune activation status. Wang et al. [13] demonstrated that none of the 12 patients who received CAR-T-cell therapy tested positive for various autoantibodies, including antinuclear antibodies and anti-double-stranded DNA antibodies, within 2 to 12 months posttreatment. Although immunoglobulin (IgG, IgA, and IgM) levels decreased within 30 d after treatment, they gradually recovered thereafter. In the study by Mougiakakos et al. [41], patients’ serum IgG concentrations remained above 5 g/l (without replacement therapy), and in the study by Wang et al. [13], salivary IgA levels returned to normal approximately 8 months later, indicating gradual restoration of immune function. The deep clearance of B cells in lymphatic and nonlymphatic tissues by CAR-T cells is key for immune reconstitution. Deep clearance lays the foundation for immune reconstitution: Taubmann et al. [53] followed patients with SLE, idiopathic inflammatory myositis, and SSc for more than 18 months, showing that CD19 CAR-T-cell therapy, through deep B-cell clearance and immune system reset, enabled all patients to meet disease remission criteria and discontinue all immunosuppressive drugs, further corroborating the central role of immune reconstitution in the durability of therapeutic efficacy. The initial expansion of CAR-T cells and dose adequacy are essential prerequisites for sustained therapeutic efficacy. In the study by Wang et al. [13], a patient who received a subtherapeutic dose of CAR-T cells experienced transient remission but failed to sustain control of autoantibodies, with recurrence even after full-dose retreatment, highlighting the importance of sufficient initial expansion. In summary, the effective expansion and sustained survival of CAR-T cells, deep clearance of B cells (in peripheral blood, lymphoid, and nonlymphoid tissues), and subsequent immune reconstitution dominated by newly generated nonautoreactive B cells collectively form the biological basis for the rapid and durable clinical remission of autoimmune diseases [54].

### Adverse reactions and safety management strategies

The adverse effects of CAR-T-cell therapy on autoimmune diseases primarily include CRS, neurotoxicity (ICANS), and immune-related adverse events. These mechanisms are associated with excessive CAR-T-cell activation, immune imbalance, and long-term functional dysregulation, necessitating targeted management to ensure safety. CRS is the most common acute adverse reaction and is associated with the release of inflammatory factors following CAR-T-cell recognition of antigens. The incidence of CRS varies substantially across different products. Among 19 patients who received NS7 CAR-T-cell therapy, 14 patients had grade 1 CRS, 4 patients had grade 2 CRS, and only 1 patient had grade 3 CRS, primarily presenting as fever, with a median onset at 1 d and a duration of 13 d. Most cases resolve spontaneously, whereas some require glucocorticoid or tocilizumab intervention [55]. Taubmann et al. [56] reported a case of severe anti-synthetase syndrome in which a patient manifested severe CRS (grade 3) and macrophage activation syndrome subsequent to CD19-targeted CAR-T-cell therapy, resulting in a critical condition. This finding underscores the possibility that CAR-T-cell therapy may induce uncontrollable immune storms within the context of autoimmune diseases. Tisagenlecleucel reported an overall CRS incidence of 63% in pediatric patients with relapsed/refractory B-cell acute lymphoblastic leukemia, with ≥grade 3 cases accounting for 21%. High disease burden (e.g., ≥5% bone marrow lymphoblasts) was a risk factor (≥grade 3 incidence: 34.7% in the high-burden group versus 9.8% in the low-burden group), with a median onset at 5 d and a duration of 4 d [57]. In 2024, Giordano Attianese et al. [58] developed an inducible-ON/OFF CAR (iONØ-CAR) (ON/OFF switch design), which enables rapid degradation of CAR proteins via lenalidomide (activation-to-inactivation transition completed in 4 to 6 h) and reversible control under venetoclax regulation, offering a new strategy for rapid CRS intervention. The incidence of ICANS is relatively low, and the mechanism may be related to the infiltration of CRS inflammatory factors through the blood–brain barrier or direct CAR-T-cell infiltration into the central nervous system. With respect to NS7 CAR-T-cell therapy, only 2 cases of grade 1 ICANS have been reported [55]; tisagenlecleucel in pediatric patients resulted in an overall incidence of neurotoxicity of 21%, with ≥ grade 3 cases accounting for 7%, a median onset time of 6 d, and a duration of 5 d. Management strategies are similar to those for CRS (tocilizumab, glucocorticoids, etc.) [57]. Controllable designs such as iONØ-CAR may further reduce this risk. Immune-related adverse events, including hypogammaglobulinemia, immune effector cell-associated hematologic toxicity, infections, and rare secondary malignancies, involve long-term immune dysregulation. CD19 CAR-T cells can deplete B cells and CD19-positive plasma cells, leading to secondary hypogammaglobulinemia, whereas immune effector cell-associated hematologic toxicity exacerbates immunodeficiency through cytopenias (anemia, neutropenia, etc.). Infections are the most common long-term adverse events: In the acute phase (<30 d), bacterial infections (catheter-related or urinary tract infections) predominate, whereas viral infections (e.g., cytomegalovirus reactivation) are more frequent in the long term (>30 d). CRS and ICANS are predictive factors for long-term infection risk [59]. Long-term follow-up (>2,205 patient-years) indicates that infections are mostly mild to moderate, with hypogammaglobulinemia being the second most common infection associated with B-cell-targeted CAR-T cells [60]. Secondary malignancies are rare: A 2025 evaluation of 783 patients revealed only 1 case (2.3%), with no evidence of vector insertional oncogenesis [60]. Management strategies include immunoglobulin replacement therapy to correct hypogammaglobulinemia, as well as early infection monitoring and prevention. Hill et al. [61] reported that hypogammaglobulinemia is the most common long-term toxicity following CAR-T-cell therapy, persisting for months to years. Immunoglobulin levels require monitoring, and when levels are excessively low, intravenous human immunoglobulin replacement therapy should be considered. Concurrently, infection prevention strategies involving antibiotics, antivirals, and antifungals should be implemented.

### Long-term efficacy monitoring and prognostic assessment methods

CAR-T-cell therapy has substantial potential in treating autoimmune diseases, making long-term efficacy monitoring and prognosis assessment critical [62]. This is achieved primarily through comprehensive approaches, including B-cell subset analysis, autoantibody monitoring, B-cell clonal analysis, and other molecular markers [2,63,64]. CD19-targeted CAR-T cells exert their effects by eliminating B cells, necessitating monitoring the depth and duration of posttreatment B-cell depletion. This finding correlates with the functional persistence of CAR-T cells and phenotypic analysis of reconstituted B cells aids in assessing the restoration of immune tolerance [4]. With the decline in the activity of CAR-T cells, patients experience B-cell reconstitution. Recent studies have demonstrated that posttreatment B cells are predominantly naive, which reflects the effective suppression of pathogenic clones and the resetting of autoimmune responses. This shift toward naive B cells suggests that the patient has likely achieved long-term remission, indicating the restoration of immune tolerance and substantial suppression of the expansion of pathogenic clones [65]. Dynamic changes in autoantibody levels—such as anti-double-stranded DNA antibodies in SLE [64] and anti-myeloperoxidase antibodies in anti-neutrophil cytoplasmic antibody-associated vasculitis [66]—serve as critical indicators for evaluating treatment efficacy and predicting relapse. B-cell clonal analysis, particularly through BCR repertoire profiling, reveals the elimination of pathogenic clones and the reconstitution of healthy clones, thereby deepening the understanding of CAR-T-cell mechanisms [67]. Additionally, cytokine profiles [67,68], CAR-T-cell persistence and function in vivo—including copy number, phenotype, and exhaustion marker expression [69]—along with overall changes in the host immune system are critical factors for evaluating long-term efficacy and safety [63]. Emerging strategies, such as CAAR-T cells and CAR-Treg cells, which are designed to more precisely regulate autoimmune responses, present new challenges and opportunities for future monitoring [67].

## Next-Generation CAR-T-Technological Frontiers and Clinical Translation Challenges

Next-generation CAR-T-cell therapies leverage novel designs, including inducible systems (such as inducible ON-CAR, which enable drug-regulated dual-switch control) and dual-target CARs (CD19/BCMA) for synergistic pathogenic cell clearance. Allogeneic universal CAR-T cells engineered via multiplex gene editing (HLA/T cell receptor [TCR] knockout) provide off-the-shelf advantages with reduced immunogenicity; however, their long-term genomic stability requires ongoing monitoring. Clinical translation faces substantial challenges, such as high manufacturing costs, limited in vivo persistence, and potential off-tumor toxicity. Future efforts should focus on cost reduction via nonviral vectors, enhanced durability through optimized CAR structures such as logic-gated designs, improved specificity via multitarget strategies, and personalized treatment on the basis of patient immune phenotypes. The safe validation of these findings necessitates large-scale longitudinal studies.

### Novel CAR design and remote control strategies

The novel CAR design and remote control strategies provide critical support for enhancing the safety, specificity, and functional regulation flexibility of CAR-T-cell therapies. In 2024, Giordano Attianese et al. [58] developed an inducible ON-CAR based on the BH3 mimetic-induced homodimerization mechanism of the Bcl-2 family protein mitochondrial apoptosis pathway: Truncated Bcl-2 proteins were integrated into the extracellular domains of the CAR receptor chain and signaling chain, enabling T-cell function activation through chain dimerization in the presence of venetoclax (activity was dose-dependent and lost within 48 h after withdrawal). Further development of an integrated CAR (iONØ-CAR) incorporated a lenalidomide-inducible degradation tag (degron) at the intracellular end of the signaling chain, achieving dual regulation through venetoclax activation and lenalidomide-induced rapid degradation (functional loss within 4 to 6 h and interferon-γ release inhibition within 24 h), with functional recovery possible after lenalidomide withdrawal. This system utilizes clinically approved small-molecule drugs and human-derived protein components, effectively reducing the risk of immunogenicity. Dual-target CAR technology substantially enhances treatment specificity and therapeutic depth by simultaneously targeting multiple disease-related antigens. Wang et al. [13] designed a BCMA-CD19 cCAR consisting of 2 independently expressed CARs targeting CD19 on B cells and BCMA on long-lived plasma cells integrated with a secreted IL-15/IL-15Rα sushi domain to increase CAR-T-cell survival. In patients with SLE accompanied by LN, this therapy achieved complete clearance of peripheral blood B cells within 1 to 10 d and recovery within 2 to 6 months; substantially reduced pathological autoantibody levels, such as anti-SSA/Ro52; improved renal function; and induced only grade 1 mild CRS, demonstrating the advantage of dual-target synergistic clearance of pathogenic cell populations. Multifunctional CAR technology expands the application potential of CAR-T cells through structural innovation and signal optimization. In terms of structural design, Whittington et al. [45] developed a novel CAR based on the HLA-DR1 molecule: Embedding the self-antigen CII peptide into the DRB1 chain, replacing the intracellular domain of DR1 with CD28 and CD3ζ signal transduction domains, and forming a functional complex on the surface of CD8^+^ T cells, which can specifically recognize and kill pathological CD4^+^ T cells presenting DR1-CII peptides. This design leverages the high-affinity binding characteristics of MHC II molecules with TCRs, differs from traditional scFv-based CAR structures, and substantially reduces disease incidence and severity in a mouse model of autoimmune arthritis.

In the realm of structural design, Whittington et al. [45] engineered a novel HLA-DR1-based CAR by integrating the CII peptide into the DRB1 chain and fusing it with the CD28 and CD3ζ intracellular domains. When expressed on CD8^+^ T cells, this CAR could specifically identify and eliminate pathological CD4^+^ T cells presenting DR1-CII peptides. Capitalizing on the high-affinity MHC II–TCR interaction, this design diverges from conventional scFv-based CARs and notably alleviated the severity of disease in an autoimmune arthritis mouse model. Spanier et al. [70] developed an InsB-g7 CAR founded on the insulin B-chain 10-23 peptide–IA*^g7^* complex. After being introduced into regulatory T cells, this CAR empowered them to recognize pancreatic autoantigens and augment suppressive function. These CAR-Treg cells completely averted diabetes in adoptive transfer models and substantially decreased the incidence of disease in spontaneously diabetic NOD mice. In terms of signal transduction optimization, Wu et al. [71] reported that single phosphorylation of the immunoreceptor tyrosine-based activation motif) in the CD3ε cytoplasmic region results in the recruitment of the inhibitory kinase Csk. Integrating this construct into a second-generation CAR construct (E28Z design) resulted in the generation of CAR-T cells with reduced cytokine release and enhanced persistence. The BRS of CD3ε further promotes cell survival through the recruitment of the phosphoinositide 3-kinase regulatory subunit p85, a strategy effective across multiple antigen targets. Zhang and colleagues [72] introduced a constitutively activated signal transducer and activator of transcription 3 (STAT3) mutant to endow CAR-T cells with both effector and memory phenotypes. This approach demonstrated sustained antitumor activity without notable off-target toxicity across multiple tumor models. This research unveils the distinctive advantage of STAT3 in regulating the function of CAR-T cells, presenting a novel strategy for optimizing cell therapies via the precise modulation of STAT signaling.

### Potential and risks of allogeneic and universal CAR-T-cell therapies

**Fig. 4.**
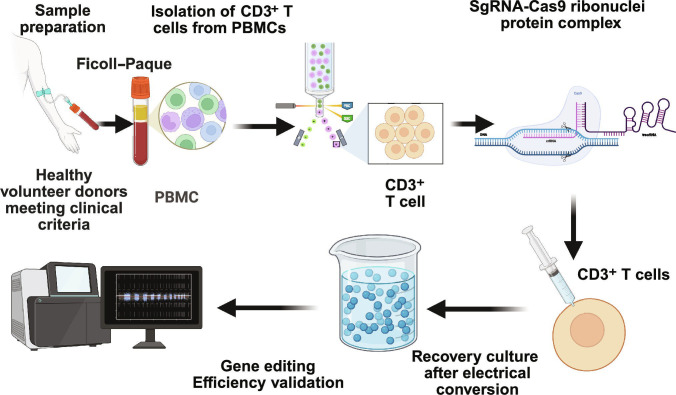
Preparation of the isogeneic product TyU19. This schematic depicts the CRISPR-Cas9 editing procedure for the preparation of TyU19 chimeric antigen receptor T (CAR-T) cells. Initially, peripheral blood is procured from healthy donors who meet clinical criteria. Peripheral blood mononuclear cells are isolated through Ficoll–Paque density gradient centrifugation, and CD3^+^ T cells are further purified from these isolated cells. A single guide RNA (sgRNA)–Cas9 ribonucleoprotein complex is subsequently constructed and introduced into CD3^+^ T cells via electroporation. After transfection, the cells are subjected to recovery culture. Ultimately, the gene editing efficiency is verified via a relevant apparatus, which comprehensively demonstrates the CRISPR-Cas9 editing process for TyU19 CAR-T cells. Created in BioRender.com.

The core advantage of allogeneic CAR-T-cell therapy lies in its universal characteristics: being prepared from healthy donors and enabling rapid off-the-shelf application (e.g., the healthy donor-derived allogeneic product TyU19) (Fig. 4), which results in high-efficiency B-cell depletion in clinical settings. Its efficacy is reflected not only in circulating B-cell depletion but also in deep tissue clearance [8]. To mitigate immune rejection and GVHD risks, allogeneic CAR-T cells typically employ multitarget gene editing strategies: TyU19 utilizes CRISPR-Cas9 technology to knock out HLA-A, HLA-B, CIITA, TRAC, and PD-1, effectively reducing the graft-versus-host potential (CD3 negativity rate exceeding 98%) while alleviating host rejection of the graft. Ex vivo and in vivo analyses revealed that gene editing targeting HLA-A/B loci induces approximately 20% off-target effects at HLA-C. However, owing to the unique immunological role of HLA-C, this does not compromise product safety and may instead reduce immunogenicity. Overall, genomic editing remains stable, with no substantial chromosomal structural abnormalities [1]. Although the immunogenicity of allogeneic CAR-T cells is reduced through gene editing, their long-term safety requires ongoing attention: While the safety risks associated with gene editing are lower than those associated with traditional approaches, their long-term efficacy and safety (including potential cumulative off-target effects and sustained genomic stability) still warrant further investigation [19]. Building on this foundation, the unique therapeutic efficacy of universal CAR-T cells in treating autoimmune diseases also stems from their potential to reshape the immune system. They not only interrupt autoimmune responses by eliminating pathogenic B cells but also promote the restoration of immune tolerance by influencing the nature of B-cell reconstitution [2,64]. This “reset” effect on the immune system holds promise for delivering more durable remissions than traditional immunosuppressive agents do, thereby reducing patients’ dependence on long-term medication and its associated side effects [64]. Furthermore, allogeneic “off-the-shelf” CAR-T-cell products offer greater accessibility to a broader patient population because of their scalable production and potential cost-effectiveness [73]. Modifying CAR-T cells through advanced gene editing technologies such as CRISPR-Cas9 can further optimize their in vivo behavior. This includes enhancing CAR-T-cell persistence and resistance to exhaustion while specifically targeting autoreactive immune cells and preserving healthy immune cell subsets. Such approaches improve treatment precision and safety [2,64].

### In vivo CAR-T-cell therapy: Paradigm shift from gene delivery to on-site modification.

Advancements in gene delivery technology have spurred the progress of in vivo CAR-T-cell generation strategies, transitioning them from the proof-of-concept stage to preclinical and early clinical trials. This transition represents a fundamental paradigm shift in cell therapy, moving from the “ex vivo preparation–reinfusion” model to the “in vivo in situ editing” model. Billingsley et al. [74] devised an antibody-conjugated liposomal nanoparticle (Ab-LNP) platform that targets extrahepatic tissues through CD3, CD5, and CD7 pan-T cell marker antibodies. This innovative combination of antibody targeting and extrahepatic LNP delivery simplifies the CAR-T-cell preparation process. It enables the temporal regulation of CAR expression via transient mRNA expression, which substantially mitigates the off-target side effects of traditional therapies. This establishes a novel technical paradigm for next-generation CAR-T-cell therapies that are safe, controllable, and widely accessible. Coradin et al. [75] developed an in vivo CAR-T-cell technology based on fourth-generation lentiviral vectors (TetraVecta) and modified Nipah virus envelopes. By incorporating a “hybrid” envelope (combining targeted and untargeted G proteins) and DARPin/VHH small-molecule targeting elements, they enhanced the transduction efficiency and specificity for CD3^+^/CD8^+^ T cells. Integration with the TRiP System to suppress CAR protein expression further reduced contamination risks and enhanced safety. Xu et al. [76] demonstrated for the first time in humans that functional CAR-T cells can be generated in vivo without ex vivo manufacturing, achieving deep remission. Despite the limited sample size and short follow-up period, this study offers preliminary evidence for the clinical feasibility of in vivo CAR-T-cell therapy. This therapy holds the potential to substantially reduce treatment costs, shorten waiting times, and improve the accessibility of CAR-T-cell therapy.

### Clinical translation challenges and future research directions

Although CAR-T-cell therapy encounters a series of translational challenges in the field of autoimmunity, the translation of this therapy into clinical application still poses a complex challenge. Nevertheless, these obstacles also define high-impact directions for future research [77]. In terms of manufacturing costs, its production heavily relies on complex automated systems to control quality and complexity. In 2021, Mougiakakos et al. [41] utilized the CliniMACS Prodigy fully enclosed automated system for CD19 CAR-T-cell production, laying the foundation for standardized manufacturing; in 2022, Mackensen et al. [5] further demonstrated that this system enables personalized CAR-T-cell expansion and purification, but high manufacturing costs and complex processes remain major obstacles to clinical translation. To address this issue, the development of nonviral vectors (such as transposon systems and CRISPR-Cas9) is considered a potential pathway to simplify manufacturing processes and reduce costs. Additionally, developing universal CAR-T cells represents another key approach to reducing preparation costs. ADI-001, developed by Adicet Therapeutics [78], is an allogeneic γδ CAR-T-cell therapy targeting CD20. In a phase I clinical trial for refractory autoimmune diseases, all 5 patients with LN achieved renal response (including 3 complete remissions), with only mild CRS (2 grade 1 cases) and no ICANS, preliminarily validating the feasibility and safety of universal CAR-T-cell therapies in autoimmune diseases. In terms of treatment durability, the survival and functional maintenance of CAR-T cells within the body are critical. In 2021, Mougiakakos et al. [41] reported that CD19 CAR-T cells rapidly expanded to 27.69% of total circulating T cells after infusion and remained detectable for more than 7 weeks. However, in 2023, Feng et al. [42] reported that peripheral blood B cells in patients began to recover approximately 3 months after CD19/BCMA CAR-T-cell therapy, suggesting potential limitations in treatment durability. In 2024, Müller et al. [9] reported that the median duration of B-cell depletion was approximately 112 d, with recovered B cells predominantly being immature or nonmemory types, and no disease relapse was observed postrecovery. Nevertheless, CAR-T-cell exhaustion may still lead to diminished efficacy, and the influence of the immune microenvironment cannot be overlooked (e.g., Qin et al. [11] reported dynamic phenotypic shifts and NK-like phenotypes in CD8^+^ CAR-T cells from IMNM patients in 2024, which may correlate with persistence), highlighting the need for further investigation into the interactions between CAR-T cells and the host immune microenvironment. With respect to antigenic precision, current CAR-T-cell regimens have already exhibited a reassuring safety spectrum in the context of autoimmune disorders [79]. However, “target-associated nondisease cytotoxicity” may still occur (i.e., CAR-T cells attack healthy cells expressing the same antigen [7]), necessitating enhanced specificity through precise target selection and CAR structure optimization. The optimization of personalized treatment strategies is key to improving efficacy and safety: Preconditioning regimens (such as fludarabine and cyclophosphamide for lymphodepletion) create a suitable immune environment for CAR-T-cell expansion and function but require dose adjustments on the basis of individual immune status; a 2024 study by Taubmann et al. [53] established a foundation for personalized therapy through standardized infusion doses (1 million CAR-T cells/kg body weight) and conditioning regimens. Future research should focus on the following directions: (a) The manufacturing costs can be reduced by exploring nonviral vectors and developing universal CAR-T cells; (b) the durability of efficacy can be extended by optimizing CAR structures (such as logic-gated designs), improving pretreatment regimens, and regulating the immune microenvironment; (c) the treatment specificity can be increased by developing multitarget CAR-T cells or CAAR-T cells, CAR-Treg, and other strategies; (d) personalized treatment should be advanced by tailoring regimens on the basis of patients’ immune phenotypes, disease subtypes, and treatment history, and long-term follow-up studies with larger sample cohorts should be conducted to validate efficacy and safety.

## Summary

Genetic engineering techniques empower the redirection of T cells toward specific surface antigens on target cells, thereby expanding the therapeutic scope of CAR-T cells beyond oncology and achieving precise immune reprogramming in autoimmune disorders [64]. This approach is fundamentally distinct from conventional immunosuppressants, which nonspecifically suppress inflammation without differentiating between pathogenic and normal immune cells. Prolonged use of such conventional immunosuppressants often leads to severe side effects, such as opportunistic infections [80]. In contrast, CAR-T-cell therapy eliminates pathogenic lymphocyte subsets while maintaining immune homeostasis, addressing the root cause of immune dysregulation [77]. In B-cell-mediated autoimmune diseases, CD19-targeted CAR-T cells effectively eliminate circulating and tissue-resident B cells and plasmablasts, while BCMA-directed CAR-T cells target long-lived plasma cells. This multitarget synergistic strategy overcomes the limitations of conventional therapies, which often fail to address dual pathogenic B-cell subsets, thus expanding the therapeutic prospects. Moreover, novel strategies, such as CAAR and CAR-Treg cells, enable targeted interventions against disease-specific pathological mechanisms, broadening the scope of applicability and enhancing safety, thereby supporting personalized therapeutic regimens. Mechanistically, CAR-T cells reestablish immune tolerance by eradicating aberrant B-cell clones, disrupting pathological lymphoid follicles, inhibiting autoantibody production, and restoring the immune microenvironment. These effects surpass the superficial anti-inflammatory actions of conventional treatments [81]. The costimulatory domains, hinge regions, and signaling modules of CAR constructs directly influence T-cell expansion, persistence, and safety. Structural optimization of these molecules can extend their in vivo lifespan and reduce adverse events, addressing the challenges of chronic medication and relapse associated with traditional therapies. Clinical data have demonstrated a positive correlation between CAR-T-cell expansion efficiency and B-cell depletion rates. Subsequently, the restoration of immune diversity occurs upon B-cell reconstitution, confirming the immune-rebuilding value of this approach. Clinically, CAR-T-cell therapy has favorable safety profiles in refractory patients unresponsive to conventional treatments. CRS and neurotoxicity predominantly present as low-grade and manageable conditions [82]. Unlike traditional therapies that require long-term maintenance and offer limited remission rates, a single infusion of CAR-T cells can induce sustained remission, accompanied by B-cell depletion and reductions in autoantibody levels. In terms of efficacy monitoring, assessing biomarkers such as B-cell subsets and autoantibody titers provides a more scientific evaluation of treatment response and prognosis compared to conventional symptom-based scoring systems. Currently, allogeneic/universal CAR-T cells are gradually moving toward industrialization. Gene editing technologies have substantially reduced the risks of GVHD and immune rejection, although long-term safety remains under surveillance. Innovative designs, such as switchable CARs, increase therapeutic controllability and hold promise for addressing the limitations of traditional treatments, including imprecise regulation and resistance associated with single-target regimens. However, high manufacturing costs and complex production processes continue to be major bottlenecks for clinical translation, necessitating breakthroughs in cell engineering and automated production platforms. Future research should focus on optimizing CAR structures, developing multitarget combinations, modulating the immune microenvironment, and evaluating long-term safety. Additionally, it is necessary to establish standardized protocols tailored to individual patient phenotypes and expand clinical data in autoimmune diseases.

In summary, CAR-T-cell therapy integrates antigen-specific targeting with precision treatment strategies, resulting in a paradigm shift from nonspecific anti-inflammatory effects to precise immune resetting, thereby offering a highly promising therapeutic approach for autoimmune diseases refractory to conventional interventions.
